# Supplementary material for: Neonatal thyroxine activation modifies epigenetic programming of the liver
Source: Nat Commun. 2021 Jul 21;12:4446. doi: 10.1038/s41467-021-24748-8 (PMC8295303; doi:10.1038/s41467-021-24748-8)
Supplement: Supplementary file 1 — Supplementary information [file 41467_2021_24748_MOESM1_ESM.pdf]

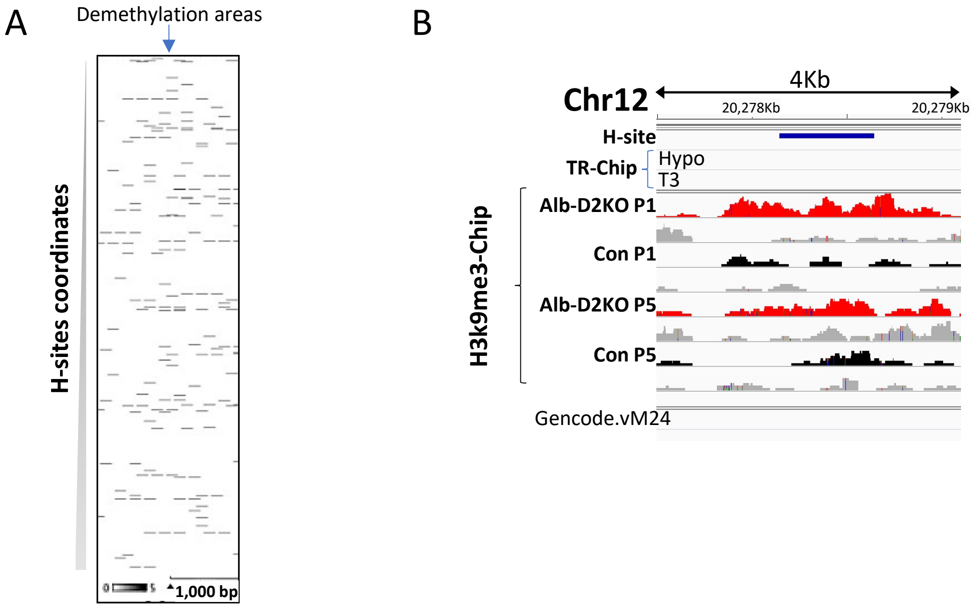

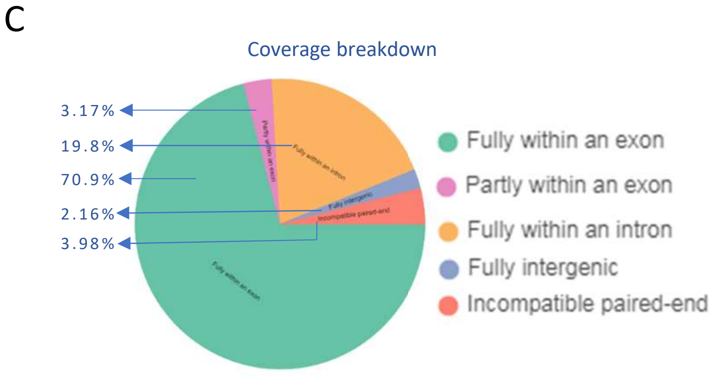

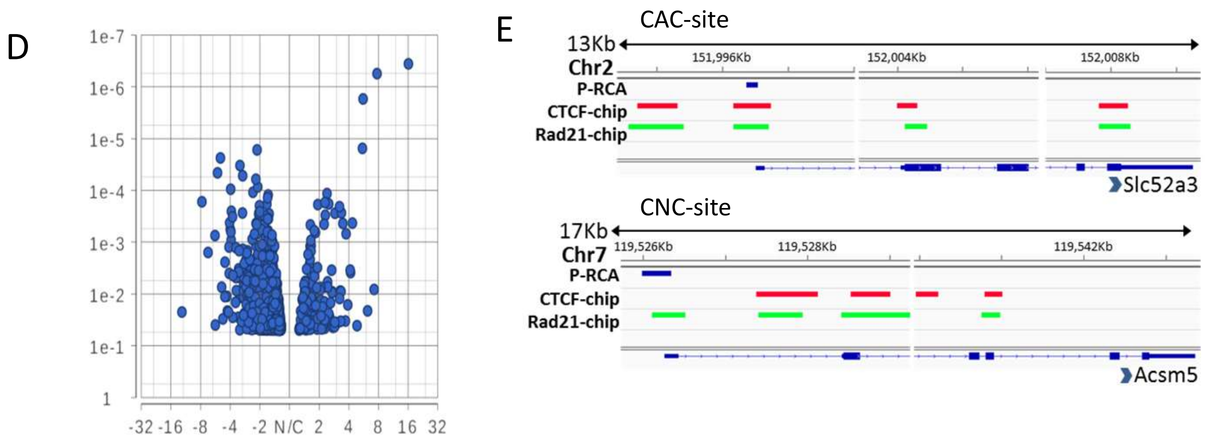

Supplementary Figure 1- (A) Heat map of H-sites coordinates vs. demethylation sites coordinates; the bottom arrow indicates 1,000 bp; the Y axis contains the H-sites coordinates, and the X axis contains the coordinates of the sites that undergo post-natal demethylation; the coordinates in the Y axis follows the chromosomal sequence: Chr 1 to Chr 9, Chr X, Chr 10 to Chr 19); (B) a close-up picture of a single H-site shown in Fig. 1A; the following tracks contain ChIP signals for: (top to bottom) H-site (blue), H3K9me3 ChIP-seq peaks in Alb-D2KO (red) and control (black) P1 and P5 mice (n=3/group); the input track is shown in gray; (C) RNA-seq coverage breakdown; distribution reflects the average of all samples; (D) differential gene expression (Volcano plot) in the Alb-D2KO vs control liver as detected by RNA-seq; each point represents the average value of one transcript in 3-4 animals. The expression difference is considered significant if p<0.05; (D) browser IGV display of two genes (Slc52a3 and Acsm5) that contain a p-RCA site (blue track); CTCF-ChIP-seq peak (red track) and Rad21-ChIP-seq peak "cohesin" (green track). Slc52a3 contains a cohesin and CTCF configuration tyopical of insulator (CAC-sites); Acsm5 contains one isolated cohesin peak (CNC-sites).

Supplementary Figure 1

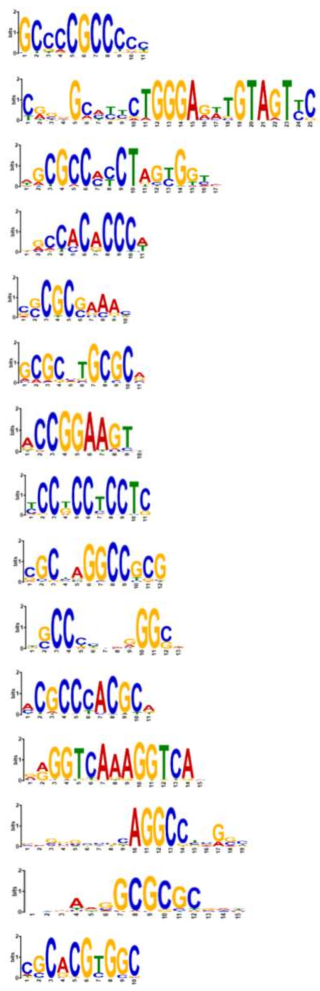

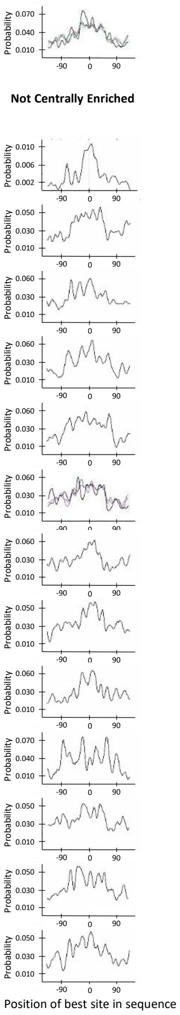

(a) Motif found	(b) Distribution	(c) Known or Similar Motifs	(d) E-value	(e) P-value
		SP1, SP1, SP3	2.9e-041	9.1e-21
	Not Centrally Enriched	ZN143, ZNF143, ZNF143	3.5e-016	
		CTCF	7.1e-013	3.2e-16
		Klf1	6.1e-010	2.8e-13
		E2F3	2.4e-007	1.1e-10
		NRF1	4.3e-007	1.9e-10
		ELK1	4.7e-007	2.1e-10
		ZNF263, SP2, SP1	3.7e-005	8.0e-6
		ZFX	9.2e-004	4.2e-7
		TFAP2A	9.6e-004	4.4e-7
		EGR2	1.0e-003	4.6e-7
		NR2F1	8.7e-003	4.0e-6
		ZFX	3.8e-002	1.7e-5
		E2F2	4.1e-005	1.9e-8
		NMYC	2.7e-002	1.2e-5

Supplementary Figure 2- Transcription factors binding motifs impoverished in the RCA areas in Alb-D2KO liver chromatin as identified through the MEME suite: (A) the logo of each motif; the relative size of the letters indicates their frequency in the sequence; the total height of the letters depicts the information content of the position in bits; (B) the distribution of the best matches to the motif in the sequences as found by a CentriMo analysis; (C) the most similar motifs reported by the motif discovery programs (MEME or CentriMo) compared with known motifs in a motif database; (D) E-value is the adjusted p-value multiplied by the number of motifs in the input files(s) and (E) p-value that is the statistical significance of the motif enrichment adjusted for multiple tests; the enrichment p-value of a motif is calculated by using the one-tailed binomial test on the number of sequences with a match to the motif ("Sequence Matches") that have their best match in the reported region ("Region Matches"), corrected for the number of regions and score thresholds tested ("Multiple Tests"). The test assumes that the probability that the best match in a sequence falls in the region is the region width divided by the number of places a motif can align in the sequence (sequence length minus motif width plus 1).

Supplementary Figure 2

Supplementary Figure 3

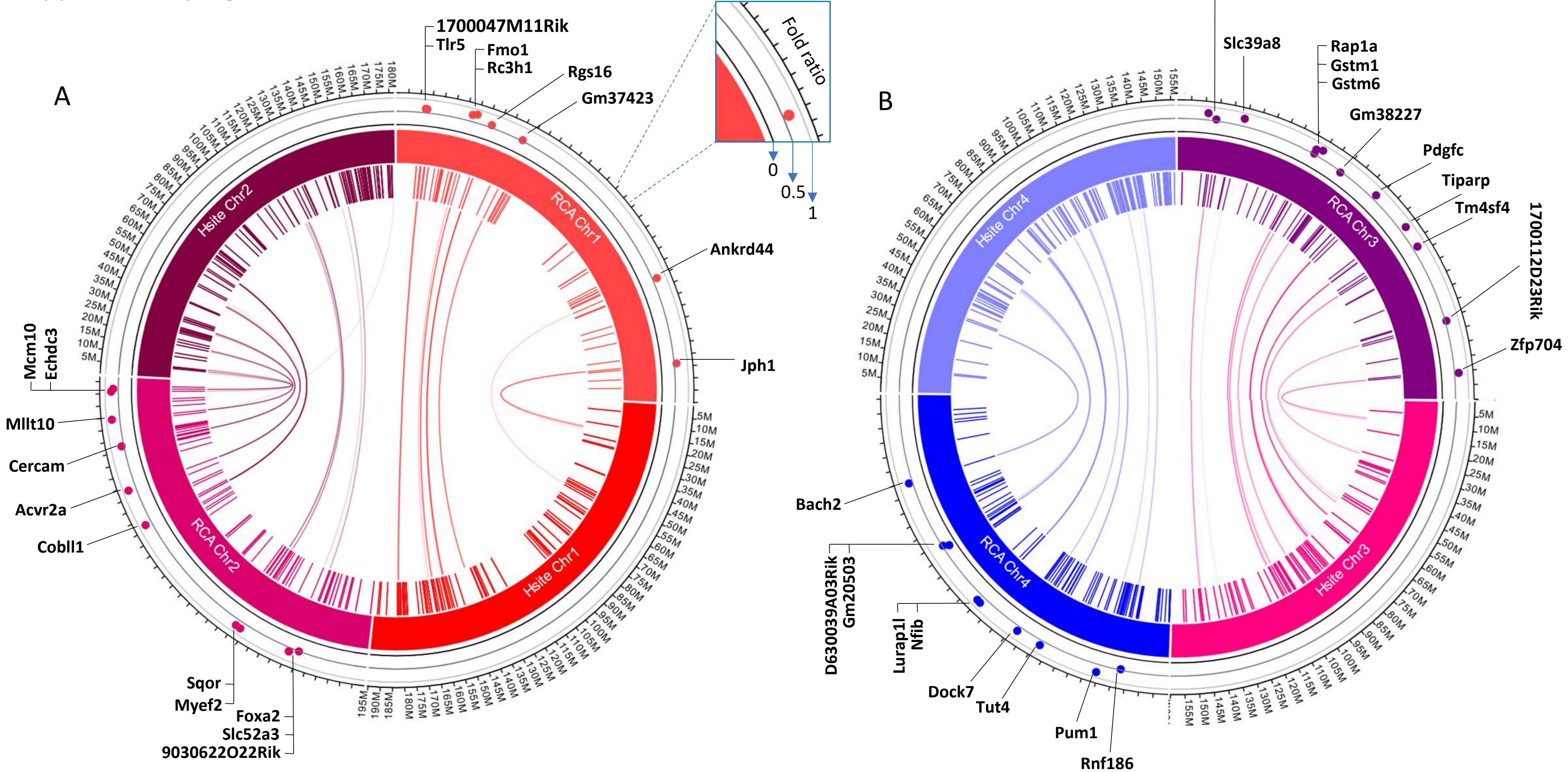

Supplementary Figure 3 (A-J) - Circa diagrams of the indicated chromosomes depicting H-sites and p-RCA areas of indicated 146 genes in which the p-RCA areas are within 5Kbp of the negative RNA-seq peaks. The outer ring (shown in the inset) is the ratio Alb-D2KO / Control gene expression: the inner black line is "0"; the dark gray line "0.5" and light gray line "1" fold-change; the location of the p-RCAs and the H-sites are indicated in the inner ring for each chromosome; the connecting lines indicate positions of areas-1 and areas-2 from Hi-C data.

Supplementary Figure 3

C

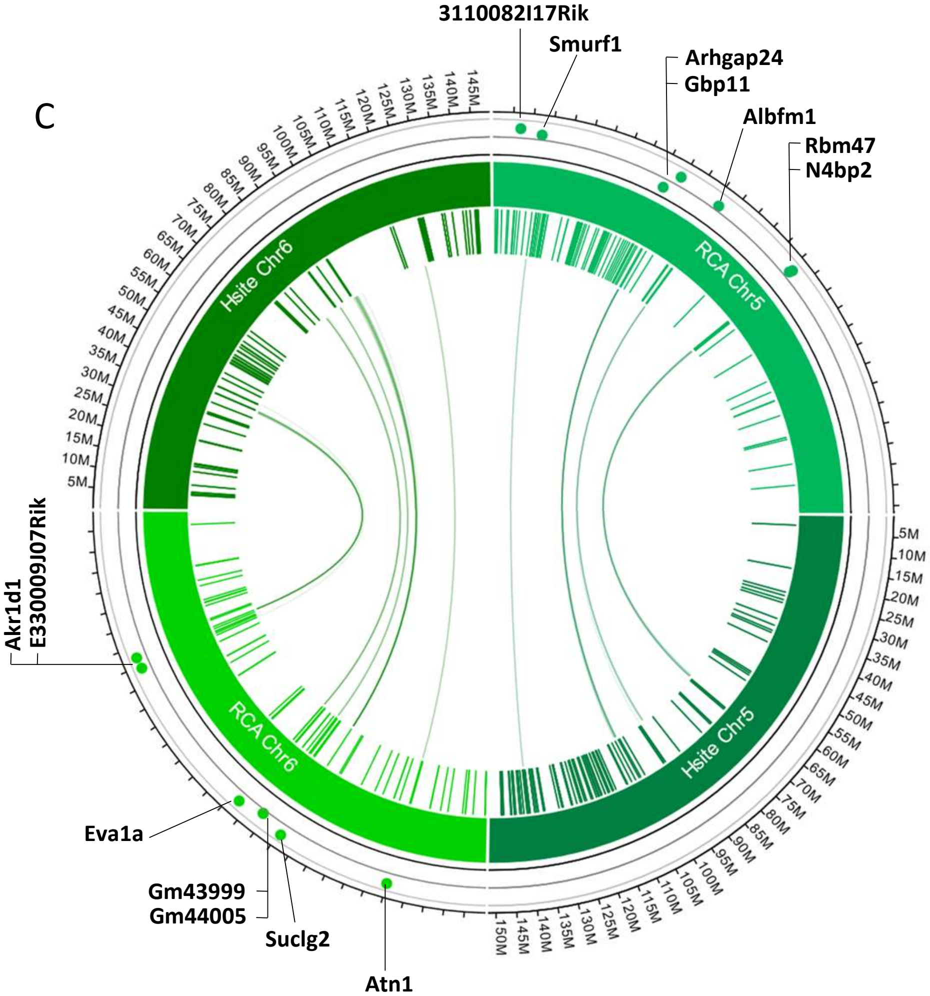

D

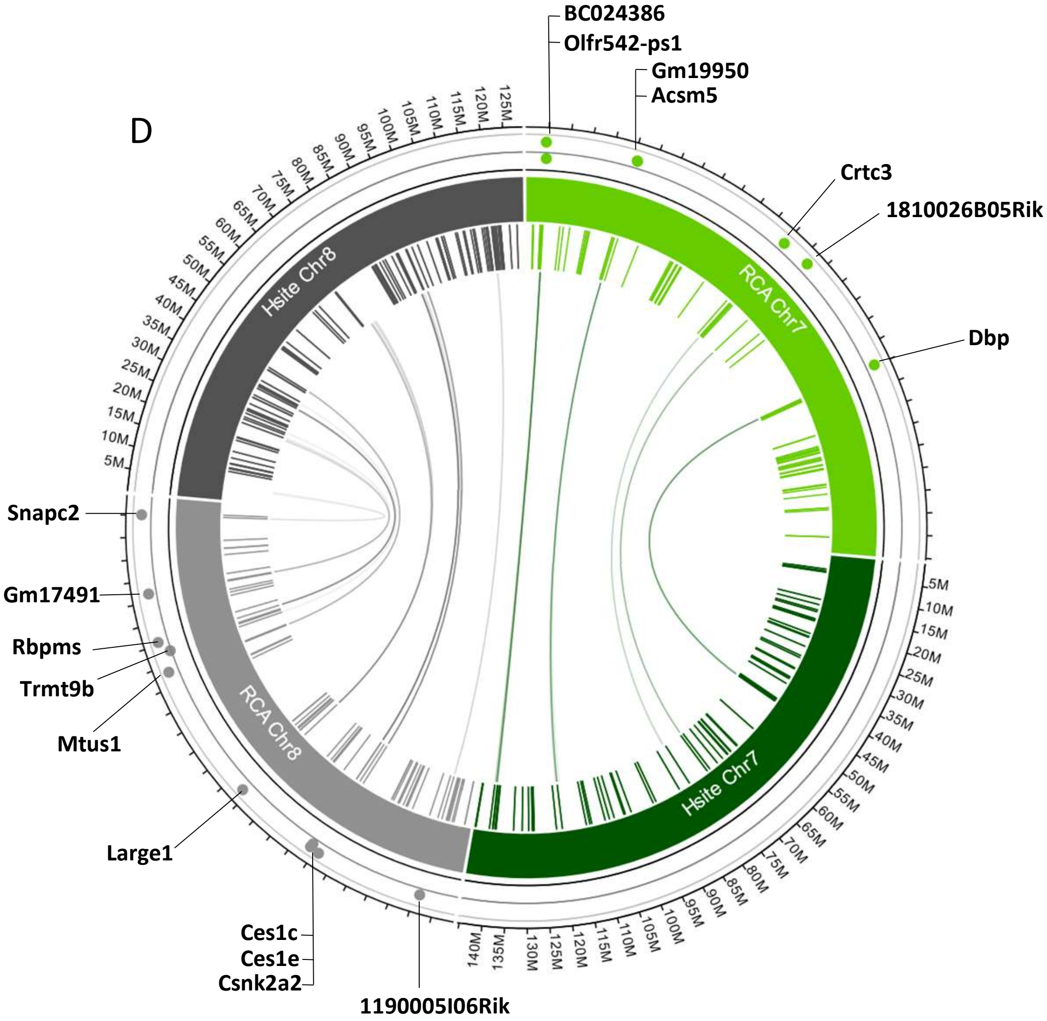

Supplementary Figure 3

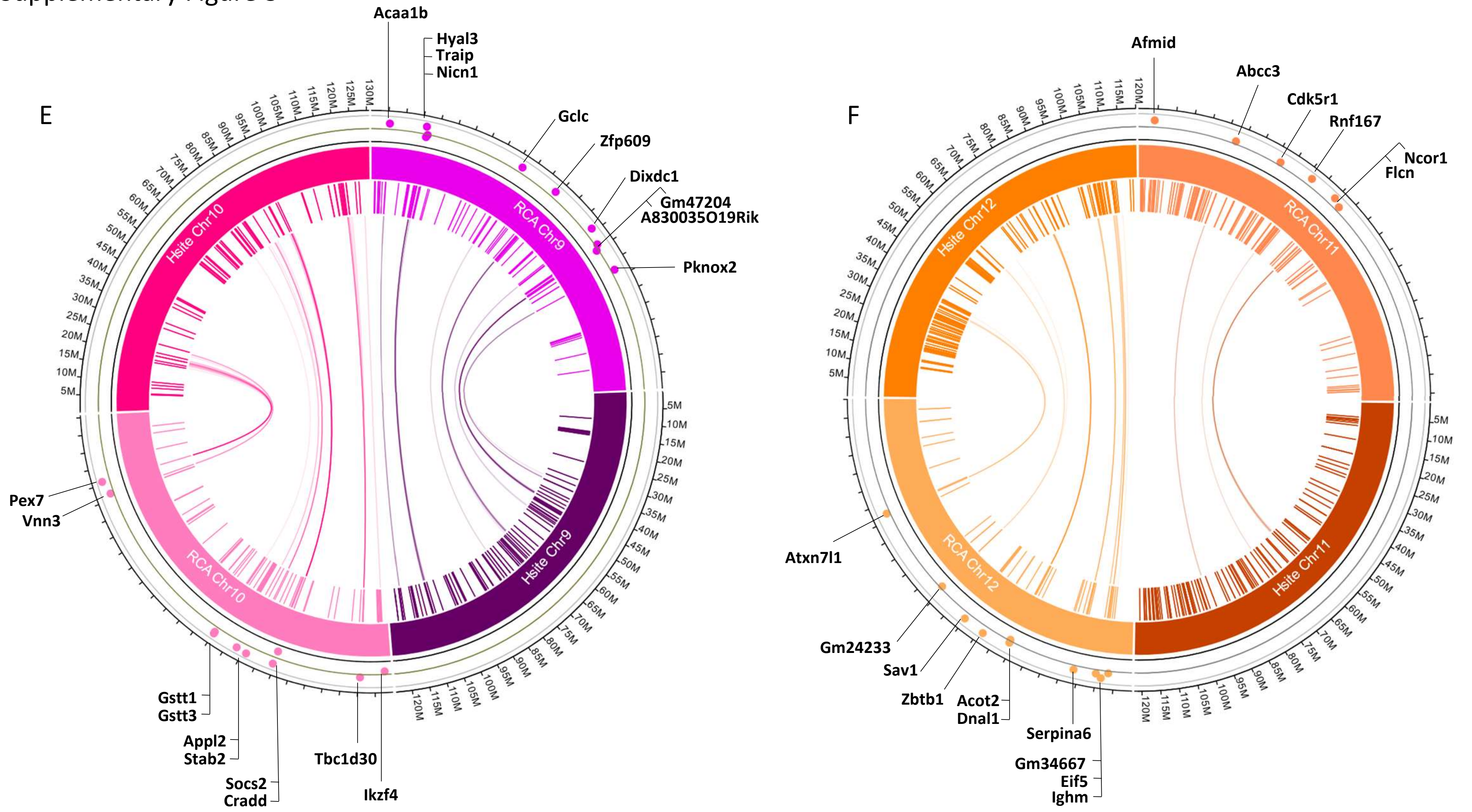

Supplementary Figure 3

G

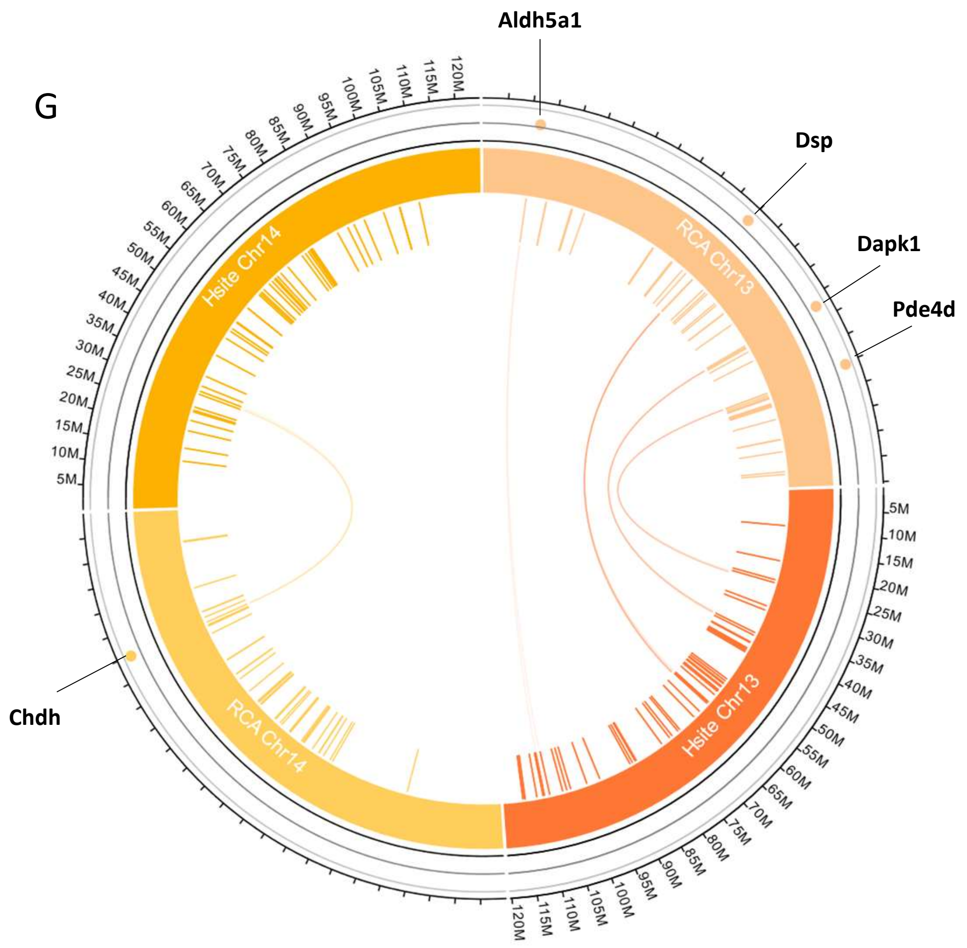

H

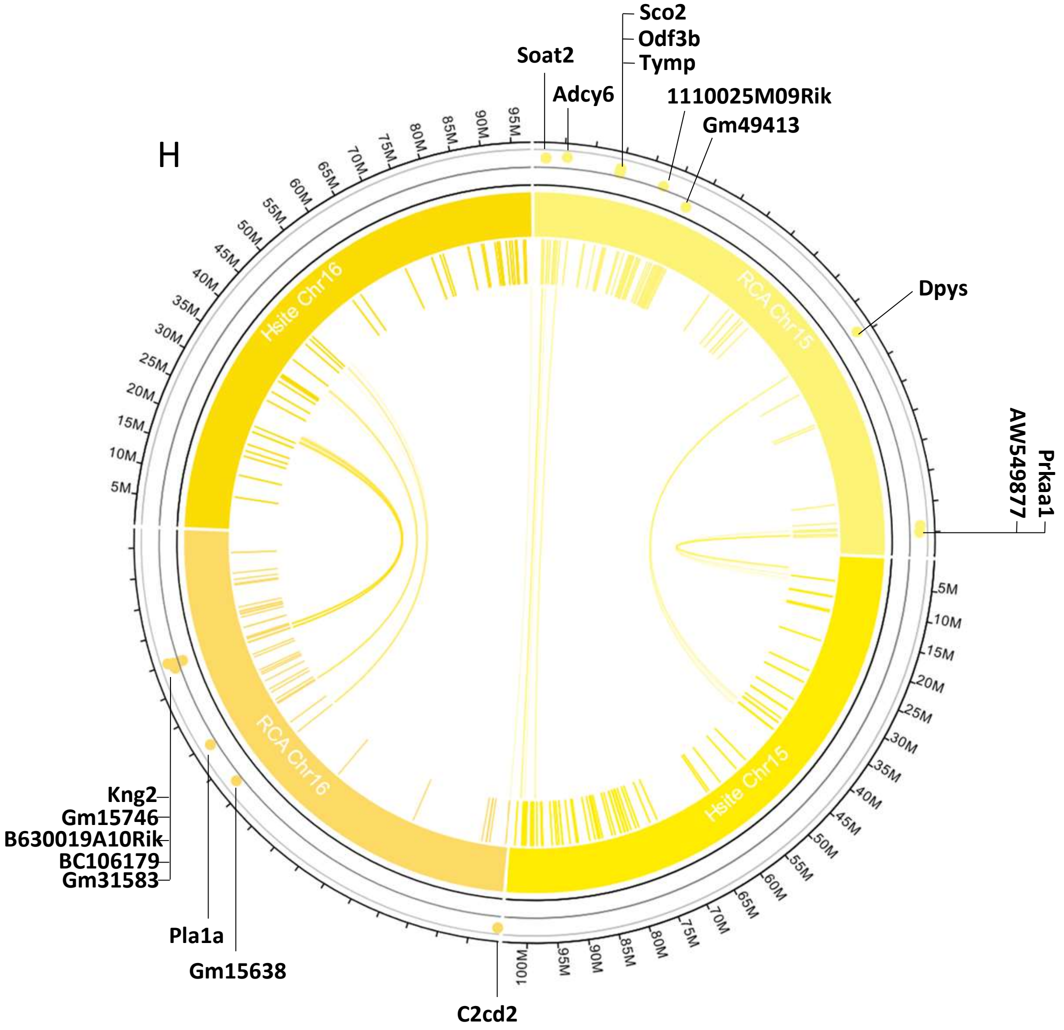

Supplementary Figure 3

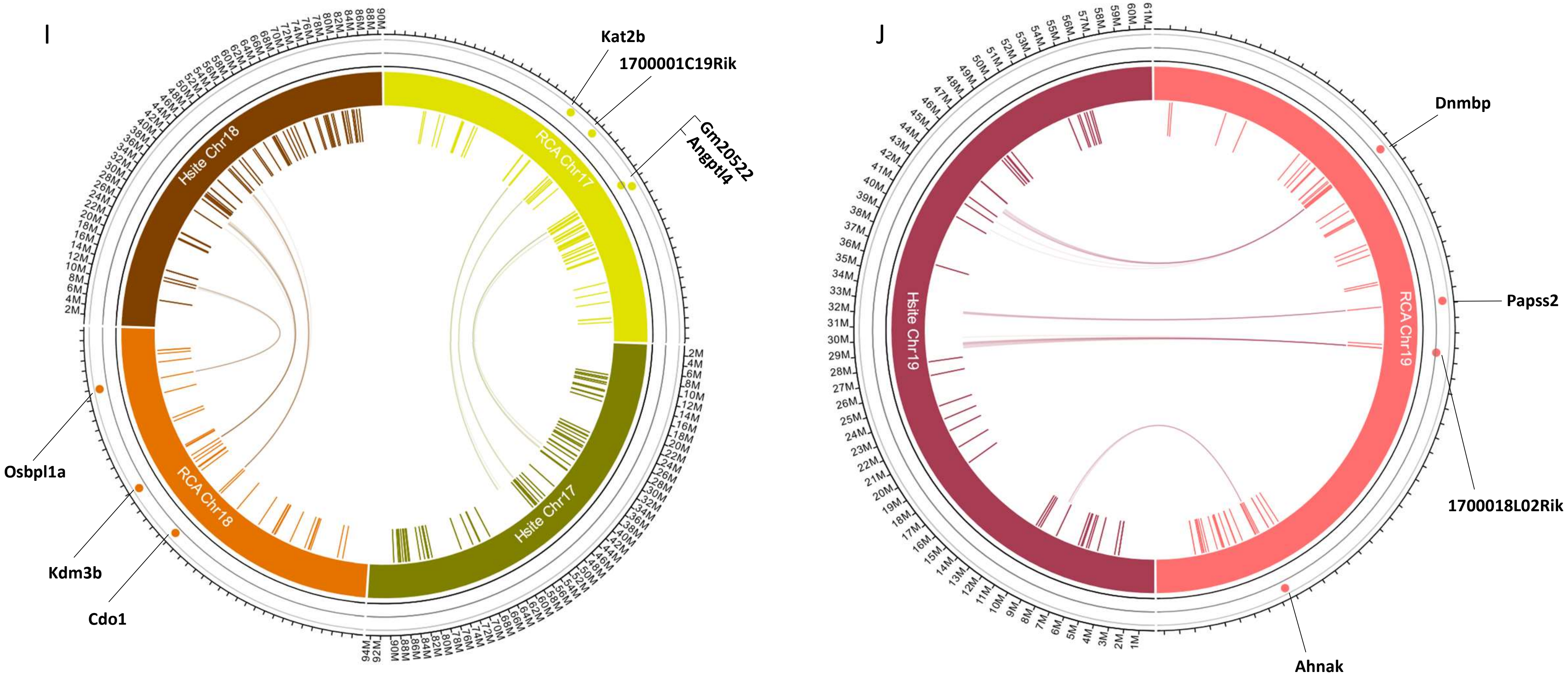

Supplementary Table 1- H-sites coordinates in Alb-D2KO mouse liver that are imbedded in areas enriched with H3K9me3. Also shown is the presence of a TR ChIP-seq peak within 10,000 bp.

**H-sites**			**H-sites imbedded in H3K9me3 area**				**H-sites flanked by TR-ChIP peaks**		
**Chromosome**	**start**	**end**	**P1**	**P5**	**P1+P5**	**Adult**	**H-site**	**uTRs**	**T3-TRs**
chr1	50386656	50387156	1	1	1	0	1	0	1
chr1	25432155	25432655	1	1	1	0	1	0	0
chr1	188902621	188903121	1	1	1	0	1	0	1
chr1	64288926	64289426	1	1	1	0	1	0	1
chr1	157803869	157804369	1	1	1	1	1	0	1
chr1	55362156	55362656	0	1	0	1	1	0	1
chr1	88222425	88222925	1	1	1	0	1	1	1
chr1	154688870	154689370	0	1	0	0	1	0	1
chr1	67097926	67098426	1	1	1	0	1	1	1
chr1	146106370	146106870	1	1	1	0	1	0	0
chr1	51747656	51748156	0	1	0	0	1	1	1
chr1	95627423	95627923	1	1	1	0	1	0	1
chr1	168879369	168879869	0	1	0	0	1	0	0
chr2	138496764	138497264	1	0	0	1	1	0	1
chr2	162142264	162142764	1	0	0	0	1	0	0
chr2	89297843	89298343	0	1	0	0	1	0	1
chr2	172058000	172058500	1	0	0	1	1	0	0
chr3	93779578	93780078	1	1	1	0	1	0	1
chr3	52415078	52415578	1	0	0	0	1	0	1
chr4	146486538	146487038	1	1	1	0	1	1	1
chr4	77040096	77040596	1	0	0	0	1	0	0
chr4	61698966	61699466	1	0	0	0	1	0	1
chr4	145449097	145449597	1	0	0	0	1	1	1
chr4	61701966	61702466	1	0	0	0	1	1	1
chr4	61715966	61716466	1	0	0	0	1	0	1
chr7	14596651	14597151	1	0	0	1	1	0	1
chr8	96151100	96151600	1	0	0	0	1	0	1
chr8	40272646	40273146	1	0	0	1	1	0	1
chr9	86902665	86903165	1	0	0	0	1	0	1
chr10	126770944	126771444	0	1	0	0	1	0	0
chr10	104933944	104934444	1	1	1	0	1	0	1
chr11	18181997	18182497	1	1	1	0	1	0	1
chr11	18182497	18182997	1	1	1	0	1	0	1
chr11	38913498	38913998	1	0	0	0	1	0	1

chr11	25856500	25857000	1	0	0	0	1	0	1
chr12	20274645	20275145	0	1	0	0	1	0	1
chr12	20267145	20267645	0	1	0	0	1	0	1
chr12	20278145	20278645	0	1	0	0	1	0	1
chr12	53220513	53221013	1	0	0	1	1	0	1
chr12	20920139	20920639	1	1	1	0	1	0	1
chr12	27136135	27136635	1	0	0	0	1	0	0
chr12	20239145	20239645	1	1	1	0	1	0	1
chr12	20264645	20265145	1	1	1	0	1	0	1
chr12	20294145	20294645	0	1	0	0	1	0	1
chr12	51492513	51493013	0	1	0	0	1	0	1
chr12	18237694	18238194	1	1	1	0	1	0	1
chr12	18328694	18329194	1	0	0	0	1	0	1
chr12	18229694	18230194	1	1	1	0	1	0	1
chr12	20281645	20282145	0	1	0	0	1	0	1
chr12	20251645	20252145	1	1	1	0	1	0	1
chr12	20845139	20845639	1	0	0	1	1	0	0
chr12	16343694	16344194	0	1	0	1	1	0	1
chr12	23267439	23267939	1	1	1	0	1	0	1
chr12	24425135	24425635	1	0	0	0	1	0	1
chr12	19199145	19199645	1	1	1	1	1	0	1
chr12	68576513	68577013	1	0	0	0	1	0	1
chr12	18243694	18244194	1	0	0	0	1	0	1
chr12	23775135	23775635	1	1	1	0	1	0	1
chr12	53221013	53221513	1	0	0	1	1	0	1
chr12	20269145	20269645	1	1	1	0	1	0	1
chr12	20284645	20285145	0	1	0	0	1	0	1
chr12	20285145	20285645	0	1	0	0	1	0	1
chr12	20291645	20292145	0	1	0	0	1	0	1
chr12	20214645	20215145	0	1	0	1	1	0	0
chr12	20293645	20294145	0	1	0	0	1	0	1
chr12	51492013	51492513	1	1	1	0	1	0	1
chr12	20219645	20220145	0	1	0	1	1	0	0
chr13	111665292	111665792	1	1	1	1	1	0	1
chr13	65311692	65312192	1	0	0	0	1	0	1
chr14	86215693	86216193	1	0	0	0	1	0	1
chr14	90987213	90987713	1	0	0	0	1	1	0
chr14	78282693	78283193	1	0	0	0	1	0	1
chr14	93899281	93899781	1	0	0	0	1	0	0
chr15	18434245	18434745	1	1	1	0	1	0	1
chr15	92696569	92697069	0	1	0	0	1	0	0

chr15	71782070	71782570	1	0	0	0	1	0	1
chr15	92750069	92750569	1	0	0	0	1	0	1
chr16	84485755	84486255	1	0	0	1	1	0	0
chr16	59284174	59284674	1	0	0	0	1	0	1
chr16	95026393	95026893	1	1	1	0	1	0	1
chr17	41079051	41079551	1	1	1	0	1	0	0
chr17	14094993	14095493	1	0	0	0	1	0	1
chr18	37341346	37341846	1	0	0	0	1	0	1
chr18	72929846	72930346	1	0	0	0	1	0	0
chr18	47646346	47646846	1	1	1	1	1	0	1
chr18	37692346	37692846	1	1	1	0	1	0	1
		**Total**	**66**	**51**	**31**	**16**	**86**	**7**	**69**

Chr is chromosome; TR is thyroid hormone receptor; uTRs is unoccupied TR, as determined in liver ChIP-seq data obtained in hypothyroid mice, present within 10,000 bp of H-site; T3-TRs is TR occupied with T3, as determined in liver ChIP-seq data obtained in T3-treated mice, present within 10,000 bp of H-site. The presence or absence of the uTR or TR ChIP-seq peak was determined by visual confirmation on the IGV browser, upstream or downstream of each H-site location.

Supplementary Table 2- Pathway enrichment analysis of differentially expressed genes in the ALB-D2KO mouse liver.

Gene set	Description	Enrichment score	P-value
path:mmu00983	Drug metabolism - other enzymes	20.5	1.22E-09
path:mmu05204	Chemical carcinogenesis	18.3	1.18E-08
path:mmu00982	Drug metabolism - cytochrome P450	17.4	2.68E-08
path:mmu00830	Retinol metabolism	15.5	1.79E-07
path:mmu00071	Fatty acid degradation	14.0	8.59E-07
path:mmu03320	PPAR signaling pathway	12.5	3.61E-06
path:mmu00980	Metabolism of xenobiotics by cytochrome P450	11.8	7.28E-06
path:mmu00140	Steroid hormone biosynthesis	11.5	9.92E-06
path:mmu00480	Glutathione metabolism	10.9	1.78E-05
path:mmu01100	Metabolic pathways	10.2	3.64E-05
path:mmu01040	Biosynthesis of unsaturated fatty acids	7.9	3.79E-04
path:mmu00590	Arachidonic acid metabolism	7.1	8.23E-04
path:mmu00591	Linoleic acid metabolism	6.5	1.48E-03
path:mmu01524	Platinum drug resistance	5.3	4.79E-03
path:mmu01212	Fatty acid metabolism	5.2	0.01
path:mmu00330	Arginine and proline metabolism	5.1	0.01
path:mmu00920	Sulfur metabolism	4.9	0.01
path:mmu00360	Phenylalanine metabolism	4.6	0.01
path:mmu00053	Ascorbate and aldarate metabolism	4.6	0.01
path:mmu00280	Valine, leucine and isoleucine degradation	4.5	0.01
path:mmu00640	Propanoate metabolism	4.5	0.01
path:mmu00040	Pentose and glucuronate interconversions	4.3	0.01
path:mmu00340	Histidine metabolism	4.2	0.02
path:mmu04979	Cholesterol metabolism	4.1	0.02
path:mmu00410	beta-Alanine metabolism	3.9	0.02
path:mmu00650	Butanoate metabolism	3.8	0.02
path:mmu00561	Glycerolipid metabolism	3.7	0.03
path:mmu04146	Peroxisome	3.6	0.03
path:mmu00380	Tryptophan metabolism	3.4	0.03
path:mmu00770	Pantothenate and CoA biosynthesis	3.3	0.04
path:mmu05418	Fluid shear stress and atherosclerosis	3.1	0.05
path:mmu05225	Hepatocellular carcinoma	3.1	0.05
path:mmu00062	Fatty acid elongation	3.0	0.05

Analysis done using Partek Flow package. Gene set column contains the KEGG pathway ID. Enrichment score: negative natural logarithm of the enrichment p-value derived from the Fisher's exact test. The higher the enrichment score, the more overrepresented the KEGG pathway is within the input list of

significant genes. An enrichment score of three is equal to a p-value of 0.05; only pathways with a $p<0.05$ are shown.

Supplementary Table 3 – Presence of CTCF and/or Cohesin ChIP-seq peaks in the p-RCA areas of the genes that contain a p-RCA within 5 Kbp of a n-RNA-seq. Genes not shown do not contain either CTCF and/or a Cohesin ChIP-seq peak.

	p-RCA coordinates					
	**chr**	**Start**	**End**	**gene location**	**CTCF**	**Cohesin**
Jph1	1	17097719	17098091	5' UTR	+	+
Rc3h1	1	160978442	160978644	3’ UTR	-	+
Fmo1	1	162866509	162866731	5' UTR	+	+
1700047M11Rik	1	182287826	182288094	5' UTR	+	+
Sqor	2	122781608	122781964	5' UTR	+	+
Slc52a3	2	151996373	151996544	5' UTR	+	+
Zfp704	3	9610026	9610292	5' UTR	+	+
Gm38227	3	96268499	96268727	5' UTR	+	+
Ddah1	3	145758456	145758830	5' UTR	+	+
D630039A03Rik	4	57916192	57916684	5' UTR	+	+
Gm20503	4	59003007	59003198	5' UTR	+	+
Lurap1l	4	80910539	80910771	5' UTR	+	+
Rbm47	5	66098169	66098540	intron	-	+
Albfm1	5	90560701	90561139	5' UTR	+	+
Gbp11	5	105346456	105346882	5' UTR	+	+
Gm44005/Gm43999	6	88742655	88743083	intron	+	+
Acsm5	7	119525990	119526343	5' UTR	-	+
Gm19950	7	119620187	119620568	intron	-	+
Olfr542-ps1	7	140717218	140717925	-	+	+
Snapc2	8	4250688	4250867	5' UTR	+	+
Ces1c	8	93131216	93131649	5' UTR	+	+
Zfp609	9	65827661	65827896	5' UTR	+	+
Gclc	9	77754387	77754633	5' UTR	+	+
Traip	9	107975388	107975651	3’ UTR	+	+
Vnn3	10	23851327	23851523	5' UTR	+	+
Stab2	10	86972034	86972224	intron	+	+
Gm24233	12	59130264	59130503	-	+	+
Serpina6	12	103657056	103657505	5' UTR	+	+
Dapk1	13	60601749	60601948	5' UTR	+	+
Kng2	16	23029015	23029479	5' UTR	+	+
Gm31583	16	24090027	24090422	5' UTR	+	+
Pla1a	16	38432879	38433347	5' UTR	+	+
Kdm3b	18	34776752	34777040	5' UTR	+	+
Stag2	X	42148924	42149305	5' UTR	+	+
				**total**	**30**	**34**

The presence “+” or absence “-“of chip-Seq peak was observed using the IGV browser. The majority of the CTCF or Cohesin peaks were located at the same position that P-RCA. Just 2 RCA sites have the CTCF/Cohesin peaks at ≤300bp of distance. RCA is reduced chromatin accessibility; CTCF is CCCTC-binding factor; **-** 3'-UTR is three prime untranslated regions and 5’UTR is five prime untranslated regions.

Supplementary Table 4- Number (upper panel) and density (lower panel) of H-sites, RCAs, p-RCAs and negative RNA-seq peaks in adult Alb-D2KO liver.

Chr #	H-sites	RCAs	p-RCAs	i-RCA	Rna-seq
1	82	416	61	140	104
2	104	551	96	182	102
3	73	379	72	135	76
4	120	608	98	179	67
5	94	517	108	133	74
6	78	463	78	155	99
7	74	463	93	141	103
8	92	453	72	137	56
9	83	454	98	140	74
10	74	440	82	133	93
11	105	687	166	205	58
12	151	319	59	113	78
13	54	315	51	115	65
14	54	307	50	84	33
15	65	413	96	112	58
16	45	253	50	58	38
17	60	383	87	108	49
18	57	237	35	72	48
19	38	321	66	77	45
X	5	40	33	6	42
Y	0	2	0	1	1

Chr #	H-sites/Chr Mbp	RCAs/Chr Mbp	p-RCAs/Mbp	i-RCA/Mbp	Rna-seq/Chr Mbp
1	0.45	2.27	0.33	0.77	0.57
2	0.62	3.26	0.57	1.08	0.60
3	0.49	2.54	0.48	0.91	0.51
4	0.86	4.34	0.70	1.28	0.48
5	0.69	3.77	0.79	0.97	0.54
6	0.57	3.36	0.57	1.12	0.72
7	0.61	3.80	0.76	1.16	0.84
8	0.77	3.81	0.61	1.15	0.47
9	0.72	3.91	0.84	1.21	0.64
10	0.61	3.64	0.68	1.10	0.77
11	0.91	5.97	1.44	1.78	0.50
12	1.44	3.04	0.56	1.08	0.74
13	0.50	2.94	0.48	1.07	0.61
14	0.50	2.87	0.47	0.79	0.31
15	0.68	4.30	1.00	1.17	0.60
16	0.49	2.78	0.55	0.64	0.42
17	0.71	4.51	1.02	1.27	0.58
18	0.68	2.82	0.42	0.86	0.57
19	0.69	5.84	1.20	1.40	0.82
X	0.04	0.30	0.25	0.04	0.31
Y	n.d.	n.d.	n.d	n.d.	n.d.

n.d. not determined; Chr is chromosome; H-sites is site of DNA methylation; RCAs is reduced chromatin accessibility area; p-RCAs is RCA area in core promoters; i-RCA is RCA area intergenic; Mbp is mega base pair.
