# Supplementary material for: Neonatal thyroxine activation modifies epigenetic programming of the liver
Source: Nat Commun. 2021 Jul 21;12:4446. doi: 10.1038/s41467-021-24748-8 (PMC8295303; doi:10.1038/s41467-021-24748-8)
Supplement: Supplementary file 3 — Description of Additional Supplementary Files [file 41467_2021_24748_MOESM3_ESM.pdf]

Description of Additional Supplementary Files

File name: Supplementary data 1
Description: H-sites chromosome coordinates and gene annotation

File name: Supplementary data 2
Description: Differentially expressed genes in adult ALB-D2KO mouse liver as compared to littermate control samples from Cre-expressing mice

File name: Supplementary data 3
Description: Gene set enrichment analysis of differentially expressed genes in the ALB-D2KO mouse liver

File name: Supplementary data 4
Description: Genes downregulated in the Alb-D2KO mouse liver that are within 5,000 bp of a p-RCA area along with their coordinates. Also included are the function of the p-RCAs, the negative transcription factor footprints identified in these p-RCAs, the annotation of the nearest H-site, the RNA-seq Alb-D2KO/control ratio and relative drop in expression

File name: Supplementary data 5
Description: Distribution of i-RCA and p-RCA across TADs within chromosome 1

File name: Supplementary data 6
Description: Distribution of i-RCA and p-RCA across TADs within 146 genes containing p-RCA:negative-RNA-seq

File name: Supplementary data 7
Description: Proximity of p-RCA, H-site and i-RCA sites to the area-2 or area-1 obtained from the Hi-C contact matrix
